# Role of Methylglyoxal in Alzheimer's Disease

**DOI:** 10.1155/2014/238485

**Published:** 2014-03-09

**Authors:** Cristina Angeloni, Laura Zambonin, Silvana Hrelia

**Affiliations:** ^1^Department for Life Quality Studies, Alma Mater Studiorum, University of Bologna, Corso d'Augusto 237, 47900 Rimini, Italy; ^2^Department of Pharmacy and Biotechnology, Alma Mater Studiorum, University of Bologna, Via Irnerio 48, 40126 Bologna, Italy

## Abstract

Alzheimer's disease is the most common and lethal neurodegenerative disorder. The major hallmarks of Alzheimer's disease are extracellular aggregation of amyloid **β** peptides and, the presence of intracellular neurofibrillary tangles formed by precipitation/aggregation of hyperphosphorylated tau protein. The etiology of Alzheimer's disease is multifactorial and a full understanding of its pathogenesis remains elusive. Some years ago, it has been suggested that glycation may contribute to both extensive protein cross-linking and oxidative stress in Alzheimer's disease. Glycation is an endogenous process that leads to the production of a class of compounds known as advanced glycation end products (AGEs). Interestingly, increased levels of AGEs have been observed in brains of Alzheimer's disease patients. Methylglyoxal, a reactive intermediate of cellular metabolism, is the most potent precursor of AGEs and is strictly correlated with an increase of oxidative stress in Alzheimer's disease. Many studies are showing that methylglyoxal and methylglyoxal-derived AGEs play a key role in the etiopathogenesis of Alzheimer's disease.

## 1. Introduction

**Figure 1:**
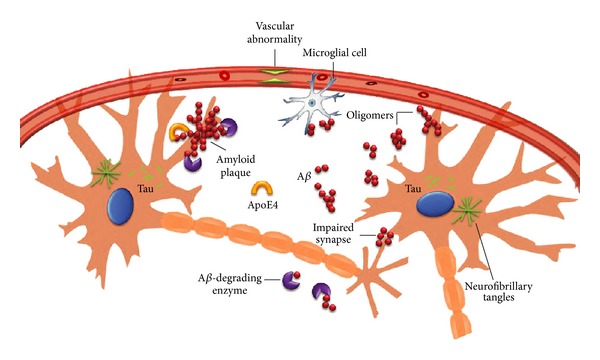
Classical processes participating in the etiology and pathogenesis of AD (modified from [131]).

Alzheimer's disease (AD) is the most common and lethal neurodegenerative disorder characterized by progressive neuronal loss and neuroinflammation in the brain and associated with progressive cognitive decline, memory impairment, and changes in behavior and personality, with rising incidence among elderly people. One of the pathological hallmarks of AD is neuritic plaques in the cerebral cortex and hippocampus. Amyloid *β* (A*β*), a 40–42 amino-acid peptide generated by proteolytic cleavages of the amyloid-*β* protein precursor (APP) [1], is one of the main components of neuritic plaques. A*β* is cytotoxic and capable of inducing oxidative stress and neurodegeneration [2, 3]. Another distinctive feature of AD is neurofibrillary tangles (NFTs), composed of bundles of paired helical filaments (PHFs) [4], mainly containing hyperphosphorylated microtubule-associated tau protein (MAP-tau) [5]. Under normal physiological conditions, tau promotes assembly and stability of microtubules and is thus involved in axonal transport [6, 7]. In AD, tau proteins aggregate forming fibrillar insoluble intracellular inclusions. The main processes involved in the etiology and pathogenesis of AD are reported in Figure 1.

The full understanding of the etiology and pathogenesis of AD has remained elusive, and more and more evidences are confirming that AD is a disease with numerous genetic and environmental contributing factors. Some years ago, it has been proposed that a chemical process known as glycation may contribute to both extensive protein cross-linking and oxidative stress in AD [8]. Nonenzymatic protein glycation is an endogenous process in which reducing sugars react with amino groups in proteins through a series of Maillard reactions forming reversible Schiff base and Amadori compounds, producing a heterogeneous class of molecules, collectively termed advanced glycation end products (AGEs) [9]. The *α*-ketoaldehyde methylglyoxal (MG), formed endogenously as a by-product of the glycolytic pathway, by degradation of triosephosphates or nonenzymatically by sugar fragmentation reactions, is the most potent precursor of AGE formation [10]. MG is able to induce cellular damage, cross-linking of proteins, and glycation [11] playing an important role in the pathogenesis of many neurodegenerative diseases [12]. In AD, AGEs accumulate in neurons and astroglia and are also found associated with neuritic amyloid plaques and NFTs [13–16]. MG may also contribute to neurodegeneration triggering oxidative stress [17–19]. Oxidative stress is characterized by an imbalance between reactive oxygen species (ROS) production and the detoxifying endogenous system. There is accumulating evidence suggesting a key role of oxidative stress in the pathophysiology of AD [20–23]. A central role for oxidative stress by the activation of NADPH oxidase in astrocytes has been demonstrated as the cause of A*β*-induced neuronal death [24] and of alterations in astrocyte mitochondrial bioenergetics that may in turn affect neuronal functioning and/or survival [25].

As oxidative stress and MG are closely interlinked, the role of MG and MG-induced production of AGEs and ROS in the development of AD is reviewed in this paper. In addition, the ability of MG to modulate detrimental redox signaling in AD has been considered.

## 2. Methylglyoxal Production

MG is a reactive intermediate of cellular metabolism, present ubiquitously in all cells. It is produced under both normal and pathological conditions via several different pathways, involving both enzymatic and nonenzymatic reactions [26]. The rate of MG formation depends on the organism, tissue, cell metabolism, and physiological conditions; therefore, MG plasma concentration reflects these factors. Plasmatic MG can be derived from exogenous sources, such as coffee, alcoholic beverages, and food [27, 28] and from endogenous sources: in situ formation in the plasma, release from cells, and loss from injured cells [29].

Since MG is ubiquitously present in living cells, almost all foods and beverages contain MG, as reviewed by Vistoli et al. [30]. The main sources of MG are represented by mono-, oligo-, and polysaccharides and lipids [31]. Several reactions and processes are involved in the accumulation of MG: autoxidation, photodegradation, and heating and prolonged storage are the main sources of MG as a degradation product in foodstuff [32–35]. Moreover, many microorganisms produce and release MG: fermentation can be a critical process increasing MG levels in alcoholic drinks and fermented foods [36]. MG is reported to originate also from environmental sources. Cigarette smoke is one of the combustion processes that can generate MG [37]; drinking water can contain MG due to the purification treatments [38]; rainwater can absorb MG from polluted air and transmits it to the soil [39].

Endogenously derived MG is formed during carbohydrate and lipid and amino acid metabolisms and involves both enzymatic and nonenzymatic reactions [40–43]. The enzymes that catalyze the reactions of MG synthesis are MG synthase, cytochrome P450 2E1, myeloperoxidase, and amino oxidase, participating in glycolytic bypass, acetone metabolism, and amino acid breakdown, respectively; nonenzymatic pathways include the spontaneous decomposition of dihydroxyacetone phosphate, the Maillard reaction, the oxidation of acetol, and lipid peroxidation [42].

The main pathway leading to MG is linked to carbohydrate metabolism and involves enzymatic and nonenzymatic degradation of the triosephosphate intermediates glyceraldehyde 3-phosphate and dihydroxyacetone-phosphate deriving from glycolysis [40, 44, 45]. It should be noted that triosephosphates originate not only from glycolytic processes but also from other routes of glucose metabolism (Entner-Doudoroff pathway, hexose monophosphate route) and from xylitol metabolism or the activity of glycerophosphate dehydrogenase, linking glycerol breakdown to MG production [40]. Dihydroxyacetone phosphate can be converted to MG by either spontaneous nonenzymatic elimination of the phosphate group or by the enzymatic contribution of MG synthase, an enzyme found in prokaryotic and mammalian systems [36, 46]. MG can also derive via the Maillard reaction in vivo under physiological conditions, similar to what is observed during food cooking and through the glycation of macromolecules and the autoxidation of carbohydrates [43].

MG production deriving from lipid metabolism is mainly linked to the acetone metabolism [47]. Acetone is derived from acetoacetate by myeloperoxidase activity and is converted to MG by the cytochrome P450 2E1 via acetol as intermediate [48]. In pathological conditions like ketosis and diabetic ketoacidosis, the oxidation of ketone bodies is likely to be an important source of MG [49]. In addition, triacylglycerol hydrolysis produces glycerol that can be transformed into MG through glycerolphosphate produced by a specific glycerol kinase [50]. Lipoperoxidation is another nonenzymatic process leading to MG formation [51, 52].

The catabolism of the aminoacids threonine and glycine (and partially tyrosine) can also generate MG through the aminoacetone intermediate [53–55]. This metabolic oxidative pathway is mediated by the enzyme semicarbazide sensitive amine oxidase (SSAO) and appears to be exacerbated in low coenzyme A states [56, 57].

## 3. Methylglyoxal Induced AGE Production

MG is able to induce protein glycation leading to the formation of AGEs [11] and is believed to be the most important source of AGEs. Glycation of proteins is a complex series of parallel and sequential reactions known as Maillard reaction [58]. Glycation starts with the reaction of glucose with lysine and leads to the formation of fructosyl-lysine (FL) and N-terminal amino acid residue-derived fructosamines while later stage reactions produce stable adducts [58]. It has been observed that FL degrades slowly to form AGEs [59] while MG reacts relatively rapidly with proteins to form AGEs [58], in particular MG is up to 20,000 times more reactive than glucose in glycation reactions [11]. MG reacts almost exclusively with arginine residues and to a lesser extent with lysine, cysteine, and tryptophan residues. The reaction of MG with arginine leads to the formation of cyclic imidazolone adducts (MG-H) [60] and other related structural isomers. MG-H is formed as three structural isomers: N*δ*-(5-hydro-5-methyl-4-imidazolon-2-yl)-ornithine (MG-H1), 2-amino-5-(2-amino-5-hydro-5-methyl-4-imidazolon-1-yl)pentanoic acid (MG-H2), and 2-amino-5-(2-amino-4-hydro-4-methyl-5-imidazolon-1-yl)pentanoic acid (MG-H3) [61]. These adducts can undergo other reactions; they can add a second MG molecule yielding either N*δ*-(4-carboxy-4,6-dimethyl-5,6-dihydroxy-1,4,5,6-tetrahydropyrimidine-2-yl)-L-ornithine (THP) [62] or argpyrimidine (N*δ*-(5-hydroxy-4,6-dimethylpyrimidine-2-yl)-l-ornithine) [63]. MG also reacts with lysine residues to form the N_*ε*_-(1-carboxyethyl)-L-lysine (CEL) and N_*ε*_-(1-carboxymethyl)-L-lysine (CML) adducts and the lysine dimer 1,3-di(N_*ε*_-lysino)-4-methyl-imidazolium (MOLD) [64]. With one lysine and one arginine residue, MG forms 2-ammonio-6-(2-[(4-ammonio-5-oxido-5-oxopentyl) amino]-4-methyl-4,5-dihydro-1H-imidazol-5-ylidene amino) hexanoate (MODIC) [65]. MG can react also with cysteine residues giving reversible hemithioacetal adducts [66] and could spontaneously modify tryptophan residues yielding *β* carboline derivatives [33].

In human serum albumin, the following concentrations of MG-derived AGEs were detected: MG-H1 2493 ± 87 mmol/mol protein; argpyrimidine 200 ± 40 mmol/mol protein; CEL 29.7 ± 1.8 mmol/mol protein; and MOL 5 ± 1 mmol/mol protein [67]. In cerebrospinal fluid of patients with amyotrophic lateral sclerosis, elevated levels of CML were reported [68], and the tissue levels of CML in cortical neurons and cerebral vessels were related to the severity of cognitive impairment in patients with cerebrovascular disease [69]. It has been demonstrated that MG is involved in the increased levels of AGEs observed in AD [70] and MG-derived AGEs such as CEL and MOLD and MG-derived hydroimidazolone have each been identified in intracellular protein deposits in neurofibrillary tangles [71] and cerebrospinal fluid [72].

## 4. Methylglyoxal Induced ROS Production

The production of ROS and reactive nitrogen species (RNS) during MG metabolism have been extensively depicted in some reviews [43, 73, 74] and a large body of literature describes the correlation among MG, AGEs, oxidative stress, and pathologies [40] such as diabetes [75], hypertension [76], aging [74, 77, 78], and neurodegeneration [13, 79].

Although the link between MG and free radicals has been investigated since the 1960s mainly by Szent-Gyorgyi [80, 81], only in 1993, the generation of ROS in a cellular system was described [82].

Free radicals and/or ROS and RNS can be produced during both the formation of MG and its degradation; the reactions involved in these processes could be summarized as follows. The enzymatic formation of MG from aminoacetone (catalyzed by SSAO) or from acetol (catalyzed by galactose oxidase) is coupled to hydrogen peroxide production [83, 84]; hydrogen peroxide is produced also when MG is converted to pyruvate by the action of the enzyme glyoxal oxidase [85, 86]. The autoxidation of aminoacetone to MG, mediated by metal ions such as Fe^2+^ and Cu^2+^, is considered a source of carbon-centered radicals and superoxide [87, 88]; similarly, the nonenzymatic reaction from acetoacetate to MG produces ROS, in the presence of myoglobin, hemoglobin, manganese, cytochrome c, or peroxidase [89, 90].

MG, likewise for monosaccharide, undergoes autoxidation [91–93] and photolysis [94], resulting in ROS generation; these reactions involve superoxide, hydrogen peroxide, and hydroxyl radical [95].

As reported in [43] and [77], ROS production related to MG has been identified in a very wide range of cellular systems, for example, vascular smooth muscle cells (VSMCs), endothelial cells, rat hepatocytes, platelet, neurons, and so forth. We have recently demonstrated that MG induces ROS production in primary culture of rat cardiomyocytes [96].

Moreover, MG is able to increase the activity of prooxidant enzymes [97–99] and to reduce antioxidants, in particular glutathione (GSH) and its enzymes [17, 100, 101]. Since the glyoxalase system that degrades MG uses reduced glutathione as a cofactor [102], decreased antioxidants in turn impair the detoxification of MG, leading to further oxidative damage.

It has been reported, furthermore, that MG can modify Cu,Zn superoxide dismutase (SOD) by covalent cross-linking, releasing copper ions from the enzyme and inactivating it [103]. Other studies indicate that MG increases mitochondrial superoxide production [104, 105].

The correlation between ROS levels and MG concentration has been reported both in animals and cultured cells [43, 76, 77]. Commonly, in cell models, the administration of MG to the medium is followed by ROS level determination, that is often obtained by the 2′,7′-dichlorodihydrofluorescein diacetate (DCFH-DA) assay or, seldom, by other tests such as lucigenin-linked chemiluminescence assay [106].

As previously reported, MG is the most reactive endogenous carbonyl able to generate AGEs. AGEs also induce oxidative stress through several mechanisms. AGEs stimulate production of cytokines and growth factors [62, 66, 107–111]. Moreover, AGEs bind to the AGE receptor (RAGE) and scavenger receptors to induce oxidative stress in various cells including VSMCs, endothelial cells, and mononuclear phagocytes [112]. In endothelial cells, AGEs increase expression of vascular cell adhesion molecule-1 (VCAM-1), intercellular adhesion molecule-1 (ICAM-1), and increase activity of nuclear factor kappa light chain enhancer of activated B cells (NF-*κ*B) to increase oxidative stress [109, 113].

## 5. Methylglyoxal and Methylglyoxal-Derived AGE Deposits in AD

As both the extracellular A*β* deposits and the intracellular NFTs have elevated stability and are long-lived proteins, they represent an ideal substrate for glycation [70]. It has been suggested that the insolubility and protease resistance of *β*-amyloid plaques are caused by extensive AGE-covalent protein cross-linking [4, 16]. In 1994, Vitek et al. observed, for the first time, that plaque fractions of AD brains contained about 3-fold more AGE adducts than preparations from healthy, age-matched controls. They showed that the in vivo half-life of *β*-amyloid is prolonged in AD, resulting in greater accumulation of AGE modifications which in turn may act to promote accumulation of additional amyloid [114]. An immunohistochemical study using a monoclonal antibody specific for AGE proteins showed extracellular AGE immunoreactivity in amyloid plaques in different cortical areas, in particular, primitive plaques, coronas of classic plaques and some glial cells in AD cortex were positive for AGEs [115]. More recently, Fawver et al. [14] stained AD brain tissue for AGEs, and similar to the previous findings, AGEs were colocalized with amyloid plaques. In addition, Ko et al. [116] showed that APP was upregulated by AGEs in vitro and in vivo, and AGEs modulate APP expression through ROS. To explore whether glycated A*β* is more toxic than authentic A*β*, Li et al. [117] treated 8-DIV embryonic hippocampal neurons with A*β* or A*β*-AGE for 24 h. They found that A*β*-AGE was more toxic than A*β* in decreasing cell viability, increasing cell apoptosis, inducing tau hyperphosphorylation, and reducing synaptic proteins. It has also been observed that MG is not only capable of increasing the rate of production of *β*-amyloid *β*-sheets, oligomers and protofibrils but also of increasing the size of the aggregates [13].

The *ε*4 allele of the apolipoprotein E (ApoE) is known as an important susceptibility gene for AD [118, 119]. It has been demonstrated that ApoE is codeposited in senile plaques in brains of patients with AD [120] and ApoE4 carriers present a higher A*β* deposition in the form of senile plaques than noncarriers [121, 122]. Interestingly, AGEs colocalized to a very high degree with ApoE and ApoE4 exhibited a 3-fold greater AGE-binding activity than the ApoE3 isoform [123]. The authors suggested that ApoE may participate in aggregate formation in the AD brain by binding to AGE-modified plaque components, which may explain why ApoE4 is associated with increased risk of AD.

As discussed above, AGEs can be localized intracellularly. Evidences have been provided that AGEs may accumulate in pyramidal neurons exhibiting a granular perikaryonal distribution in human brain whereas animals show a nuclear staining pattern [124]. It has been shown that AGEs accumulate in endosomal and lysosomal vesicles of pyramidal neurons in the hippocampus, the dentate gyrus, cortical layers III, V, and VI, and in entorhinal cortical layers II, III, V, and VI [125]. Interestingly, Wong et al. [126] observed colocalization of AGEs and inducible nitric oxide synthase (iNOS) in a few astrocytes in the upper neuronal layers in the early stage AD brains, while, in late AD brains, there was a much denser accumulation of astrocytes colocalized with AGEs and iNOS in the deeper and particularly upper neuronal layers. An immunohistochemical study showed that, in AD patients, the percentage of AGE-positive neurons (and astroglia) increases with the progression of the disease and those neurons which show diffuse cytosolic AGE immunoreactivity also contain hyperphosphorylated tau, suggesting a link between AGE accumulation and the formation of early neurofibrillary tangles [16]. Using specific AGE antibodies directed against CML, pyrraline, and hexitol-lysine it has been demonstrated that AGEs are colocalized with NFTs [15, 127, 128].

In AD patients, AGEs accumulate also in the cerebrospinal fluid (CSF), which is in close contact with the brain. An increased accumulation of Amadori products in all major proteins of CSF of AD patients including albumin, apolipoprotein E, and transthyretin has been observed [129]. Bär et al. [130] measured significantly elevated levels of CML in CSF of AD patients when compared to controls. In CSF protein, Ahmed et al. [72] observed an increased levels of CML residues in subjects with AD and in CSF ultrafiltrate; the concentrations of MG-derived hydroimidazolone free adducts were also increased.

## 6. Role of Methylglyoxal and Methylglyoxal-Derived AGEs in the Progression of AD

The process underlying AD is complex and involves many different features such as mitochondrial dysfunction, abnormal protein aggregation, inflammation, and excitotoxicity. Beeri et al. [132] conducted an interesting clinical study on 267 subjects, at least 75 years old, and cognitively intact at the beginning of the project. They demonstrated that the subjects with higher serum levels of MG had a faster rate of cognitive decline. Several potential mechanisms have been suggested to explain MG and MG-derived AGE neurotoxicity. Krautwald and Münch [70] suggested that AGEs contribute to the pathogenesis of AD in two different ways: cross-linking cytoskeletal proteins inducing neuronal dysfunction and death and accumulating on A*β* deposits chronically activating micro- and astroglial cells, as widely underlined in the previous paragraph. Moreover, it has been observed that MG is a neurotoxic mediator of oxidative damage in the progression of AD and other neurodegenerative diseases [133]. The brain is highly susceptible to oxidative stress due to its high energy demand, high oxygen consumption, large amounts of peroxidizable polyunsaturated fatty acids, and low levels of antioxidant enzymes [134]. It is no wonder that ROS induced damage to biomolecules is widely reported in AD and increasing evidences suggest that oxidative stress plays a critical role in the disease [135]. As the impairment of mitochondrial function is the main source of ROS generation and also a major target of oxidative damage, mitochondrial dysfunction has been implicated in AD [136, 137]. de Arriba et al. [138] demonstrated that MG may seriously affect mitochondrial respiration and the energetic status of cells. In particular, they observed that MG increases intracellular ROS and lactate production in SH-SY5Y neuroblastoma cells and decreases mitochondrial membrane potential and intracellular ATP levels. SH-SY5Y neuroblastoma cells have been extensively used to study the effect of MG as they show greater sensitivity to MG challenge, due to a defective antioxidant and detoxifying ability [17]. Huang et al. [139] observed that MG induced Neuro-2A neuroblastoma cell line apoptosis via alternation of mitochondrial membrane potential and Bax/Bcl-2 ratio, activation of caspase-3, and cleavage of poly(ADP-ribose) polymerase (PARP). Moreover, they investigated the mechanisms behind MG-induced neuronal cell apoptosis demonstrating that MG activates proapoptotic mitogen-activated protein kinase (MAPK) signaling pathways (JNK and p38). This data is in agreement with the results of Chen et al. [140] that, using primary cultures of rat hippocampal neurons, demonstrated that MG increases the expression level of cleaved caspase-3 and decreases Bcl-2/Bax ratio. As activated caspase-3 immunoreactivity is elevated in AD and exhibits a high degree of colocalization with NFTs and senile plaque in AD brain, it has been suggested that activated caspase-3 may be a factor in functional decline [63].

AGEs exert direct toxicity to cells through predominantly apoptotic mechanisms. Yin et al. [141] investigated the effects of AGEs in SH-SY5Y cells and rat cortical neurons. They observed that AGEs induce cell death increasing intracellular ROS through the increase of NADPH oxidase activity. Moreover, endoplasmic reticulum stress was triggered by AGE-induced oxidative stress, resulting in the activation of C/EBP homologous protein (CHOP) and caspase-12 that consequently initiates cell death. Tau phosphorylation is strictly controlled by the coordinated activities of tau phosphatase(s) and tau kinase(s), and the hyperphosphorylation of tau in the AD brain might be due to the overactive protein kinases and/or inactivation of protein phosphatases [142, 143]. Tau can be phosphorylated by different protein kinases such as the members of the MAPK family (JNK, p38 and Erk1/2), GSK-3*β*, and cyclin-dependent kinase 5 (cdk5), while protein phosphatase (PP) 2A plays a major role in regulating dephosphorylating of the hyperphosphorylated tau isolated from the AD brains [143–147]. Using wild-type mouse N2a cells, Li et al. [148] observed that MG induces tau hyperphosphorylation and activates GSK-3*β* and p38, while the simultaneous inhibition of GSK-3*β* or p38 could attenuate MG-induced tau hyperphosphorylation, suggesting an important roles of GSK-3*β* and p38 in the MG-induced NTFs formation. On the other hand, an interesting proteomic study demonstrated a decreased level of PP2 in SH-SY5Y cells subjected to MG-induced oxidative stress. Thus, it could be speculated that MG has a double role in inducing tau hyperphosphorylation: enhancing kinase activities and reducing phosphatase level. Besides hyperphosphorylation, it has been suggested that carbonyl-derived posttranslational modifications of neurofilaments may account for the biochemical properties of NFTs, likely as a result of extensive cross-links [149, 150]. Kuhla et al. [151], in an in vitro experiment, incubated wild-type and seven pseudophosphorylated mutant tau proteins with MG and observed the formation of PHF-like structures. Interestingly, MG formed PHFs in a concentration-dependent manner and this process could be accelerated by hyperphosphorylation.

## 7. Redox Signaling Modulated by Methylglyoxal in AD

As previously highlighted, MG cytotoxicity to tissue or cells is mainly mediated through an increase of oxidative stress and an induction of apoptosis. Oxidative stress is thought to play a causative role in the development of AD [152, 153]. Such stress is a typical activator of two important MAPK pathways in AD: the JNK and the p38 signaling pathways [154]. It has been suggested that the activation of the MAPK signaling pathways contributes to AD pathogenesis through different mechanisms including induction of apoptosis in neurons [155–158], activation of *β*- and *γ*-secretases, [159, 160] and phosphorylation and stabilization of APP [161, 162]. Different studies have associated MG with MAPK pathways. In RAW 264.7 cells, MG stimulated the simultaneous activation of p44/42 and p38 MAPK and also stimulates the translocation to the cell membranes of another important protein kinase involved in cellular signaling: protein kinase C (PKC) [163]. Moreover, Pal et al. [164] indicated that MG stimulates iNOS activation by p38 MAPK-NF-*κβ*-dependent pathway and ROS production by ERK and JNK activation in sarcoma-180 tumor bearing mice.

Regarding the implications of MAPK signaling pathway in oxidative damage leading to apoptosis, it has been observed that MG is able to induce apoptosis in PC12 cells through the phosphatidylinositol-3 kinase/Akt/mammalian target of rapamycin/gamma-glutamylcysteine ligase catalytic subunit (PI3K/Akt/mTOR/GCLc)/redox signaling pathway. Huang et al. [165] indicated that MG-induced Neuro-2A cell apoptosis was mediated through activation of the MAPK signaling pathway mediated by p38 and JNK. Recently, Heimfarth et al. [166] demonstrated that the exposure of slices of cerebral cortex and hippocampus of new born rats to mM MG induced ROS production and cytotoxicity. In particular, they showed that the signaling pathway mediated by ERK is totally implicated in the ROS-mediated cytotoxic damage as the initial blockage of MEK/ERK signaling pathway might be useful for the protection of cells from the high ROS levels. Additionally, they observed that p38MAPK and JNK pathway activation is related with ROS-independent mechanisms leading to reduced cell viability and apoptotic cell death.

Moreover, as it has been underlined in the previous paragraph, the MG activation of GSK-3*β* and p38 MAPK induces AD tau hyperphosphorylation [148].

## 8. Conclusions

**Figure 2:**
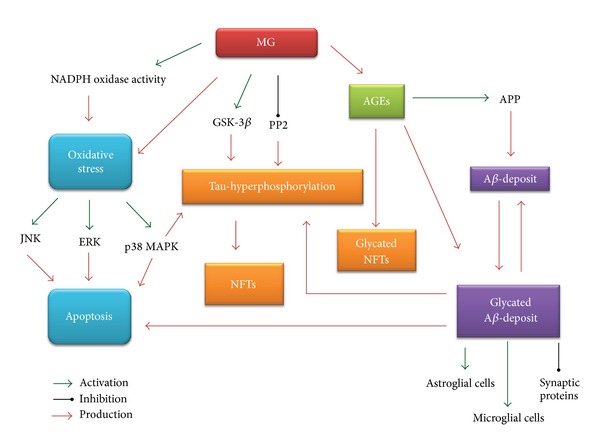
Role of MG and MG-derived AGEs in AD.

Many scientific evidences revealed different important actions of MG on signal transduction, redox balance, and cell energetic status as well as homeostatic control of cellular function. Elevated MG levels induce AGEs and ROS production playing a role in AD by several mechanisms (Figure 2). AGEs extensively cross-link proteins in A*β* deposits and neurofilaments exacerbating AD pathological hallmarks. In particular, AGEs cross-link proteins in A*β* deposits making them more insoluble, protease resistant, and more toxic. MG induces tau hyperphosphorylation by enhancing kinase activities and reducing phosphatase level. Moreover, MG is a neurotoxic mediators of oxidative stress in the progression of AD and is capable of activating many redox signaling pathways leading to apoptosis and cellular dysfunction. Accumulation of AGEs further magnifies ROS production by inducing the glycation of important antioxidant enzymes and by providing precursor of oxidative stress. In conclusion, it can be reasonably supposed that cognitive decline associated with AD might be strongly linked to an increase in MG levels due to an oxoaldehyde detoxification impairment or an altered endogenous oxoaldehyde production. From a clinical point of view, the reduction of risk factors for pathologies such as diabetes, characterized by MG accumulation due to hyperglycemic conditions and impaired glucose metabolism [167], and the enhancement of MG scavenging system may provide new therapeutic opportunities to reduce the pathophysiological modifications associated with carbonyl stress in AD.

## Abbreviation List

AD: Alzheimer's disease
AGEs: Advanced glycation end products
ApoE: Apolipoprotein E
APP: Amyloid-*β* protein precursor
Argpyrimidine: N*δ*-(5-Hydroxy-4,6-dimethylpyrimidine-2-yl)-l-ornithine
A*β*: Amyloid *β*
cdk5: Cyclin-dependent kinase 5
CEL: N*ε*-(1-Carboxyethyl)-L-lysine
CHOP: C/EBP homologous protein
CML: N*ε*-(1-Carboxymethyl)-L-lysine
CSF: Cerebrospinal fluid
DCFH-DA: 2′,7′-Dichlorodihydrofluorescein diacetate
FL: Fructosyl-lysine
GSH: Glutathione
ICAM-1: Intercellular adhesion molecule-1
iNOS: Inducible nitric oxide synthase
MAP-tau: Microtubule-associated tau protein
MAPK: Mitogen activated protein kinase
MG-H: Imidazolone adducts (methylglyoxal-derived hydroimidazolone)
MG-H1: N*δ*-(5-Hydro-5-methyl-4-imidazolon-2-yl)-ornithine
MG-H2: 2-Amino-5-(2-amino-5-hydro-5-methyl-4-imidazolon-1-yl) pentanoic acid
MG-H3: 2-Amino-5-(2-amino-4-hydro-4-methyl-5-imidazolon-1-yl) pentanoic acid
MG: Methylglyoxal
MODIC: 2-Ammonio-6-(2-[(4-ammonio-5-oxido-5-oxopentyl) amino]-4-methyl-4, 5-dihydro-1H-imidazol-5-ylidene amino) hexanoate
MOLD: 1,3-Di(N*ε*-lysino)-4-methyl-imidazolium
NADPH: Nicotinamide adenine dinucleotide phosphate
NF-*κ*B: Nuclear factor kappa light chain enhancer of activated B cells
NFTs: Neurofibrillary tangles
PARP: Poly (ADP-ribose) polymerase
PHFs: Paired helical filaments
PI3K/Akt/mTOR/GCLc: Phosphatidylinositol-3 kinase/Akt/mammalian target of rapamycin/gamma-glutamylcysteine ligase catalytic subunit
PKC: Protein kinase C
PP: Protein phosphatase
RAGE: Receptor for AGEs
RNS: Reactive nitrogen species
ROS: Reactive oxygen species
SOD: Superoxide dismutase
SSAO: Semicarbazide sensitive amine oxidase
THP: N*δ*-(4-Carboxy-4,6-dimethyl-5,6-dihydroxy-1,4,5,6-tetrahydropyrimidine-2-yl)-L-ornithine
VCAM-1: Vascular cell adhesion molecule-1
VSMCs: Vascular smooth muscle cells.
